# Comparison of Tagging Technologies for Safeguards of Copper Canisters for Nuclear Spent Fuel

**DOI:** 10.3390/s18040929

**Published:** 2018-03-21

**Authors:** Chiara Clementi, François Littmann, Lorenzo Capineri

**Affiliations:** 1 European Commission, Joint Research Centre, I-21027 Ispra, Italy; chiara.clementi@ec.europa.eu; 2 Department of Information Engineering, University of Florence, 50139 Florence, Italy; lorenzo.capineri@unifi.it

**Keywords:** identification tags, nuclear spent fuel, copper canisters

## Abstract

Several countries are planning to store nuclear spent fuel in long term geological repositories, preserved by copper canisters with an iron insert. This new approach involves many challenging problems and one is to satisfy safeguards requirements: the Continuity of Knowledge (CoK) of the fuel must be kept from the encapsulation plant up to the final repository. To date, no measurement system has been suggested for a unique identification and authentication. Following the list of the most important safeguards, safety and security requirements for copper canisters identification and authentication, a review of conventional tagging technologies and measurement systems for nuclear items is reported in this paper. The aim of this study is to verify to what extent each technology could be potentially used for keeping the CoK of copper canisters. Several tagging methods are briefly described and compared, discussing advantages and disadvantages.

## 1. Introduction

**Figure 1:**
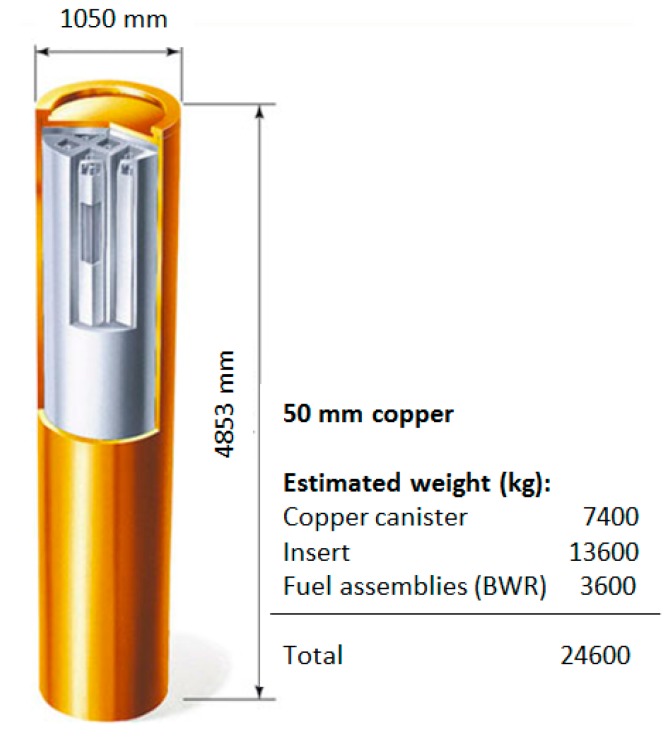
Copper canisters assembly with carbon steel containers for spent nuclear fuel.

The spent fuel generated from the operation of nuclear reactors is still highly radioactive and will continue to generate high heat levels for years. Therefore, after removal from the reactor core, the fuel needs to be safely managed in two alternative ways: reprocessing (when the fuel is seen as a potential future energy resource) or the direct storage (when the fuel is considered a waste). Either management options will involve a number of steps, which will necessarily include storage of the spent fuel for some period of time. Storage options include wet storage, in some form of storage pool, or dry storage, in a facility or storage casks built for this purpose. Over the years a number of different designs for both wet and dry storage have been developed and used in different countries [1]. However, the need to keep long-lived radioactive wastes (including spent nuclear fuel) contained and isolated from humans and the environment for a very long time, has led to the idea of long term disposal in underground repositories. The ‘multi-barrier’ concept provides isolation by a combination of engineered and natural barriers (rock, salt, clay) and no obligation to actively maintain the facility is passed on to future generations. Several countries have already invested many years and resources toward implementing final disposal of spent nuclear fuel in a geological repository [2]. Among them, Sweden is one of the countries in which this process is most advanced. The Swedish Nuclear Fuel and Waste Management Co. (SKB) developed the method for the final disposal of the spent nuclear fuel at a depth of about 500 m underground in Swedish bedrock. The fuel will be placed in canisters with an external shell made of copper and an insert of cast iron (Figure 1). These canisters will be surrounded by a special kind of bentonite clay that swells when it comes in contact with groundwater. Copper endures the anoxic conditions in the deep bedrock and the initially 50 mm thickness of the copper shell ensures that the waste will be safely contained for the time required [3,4,5]. The entire storage will ultimately be filled with bentonite clay, keeping the fuel isolated from human beings and the environment. 

**Figure 2:**
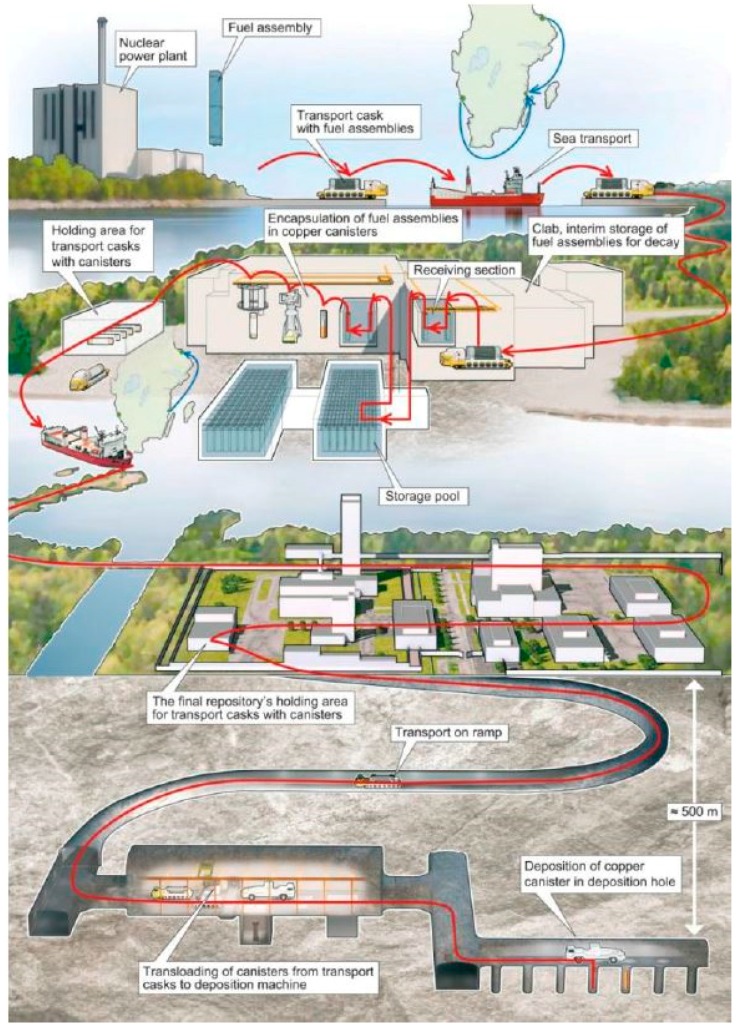
Spent nuclear fuel path along facilities. Source: SKB.

The final repository shall be designed so that it does not require maintenance or supervision. The reason for this is that there is no way to guarantee supervision and maintenance over a period of time lasting perhaps many thousands of years [6]. The SKB’s method for final disposal of fuel comprises a number of facilities that together provide a safe chain (Figure 2). The fuel coming from Swedish reactors is stored for one year minimum in the spent fuel ponds at the reactors, before it is shipped to the interim storage facility. There, the fuel is placed in storage baskets which are stored in ponds for several decades (up to 40 years). During this time, the thermal and ionizing radiation decreases significantly. Later, storage canisters with spent fuel will be lifted from the pools and moved to the encapsulation plant where the fuel is inserted in copper canisters with iron inserts. After encapsulation, the canisters are transported to the final repository and then located in the deposition hole. About 6000 copper canisters will be deposited, with an average of one canister per day [7].

During transport and deposition of canisters, from the encapsulation plant to the final repository, it is extremely important to identify uniquely each container in order to guarantee the Continuity of Knowledge (CoK) of spent nuclear fuel. CoK is defined as, the outcome of ‘a system of data or information regarding an item or activity that is uninterrupted and authentic and provides the International Atomic Energy Agency (IAEA) and the European Atomic Energy Community (EURATOM)with adequate insight to draw conclusions that nuclear material is not being diverted from peaceful purposes’ [8]. Maintaining the CoK of spent nuclear fuel throughout its life cycle means then deploying the most robust containment and surveillance (C/S) measures [9].

Concerning copper canisters, the IAEA safeguards approaches propose to use canister identification to support the CoK. SKB has to date not presented any method for labelling the copper canisters [7]. An external engraving or marking of the canister should be avoided because it may impede the long term safety and integrity of the container since it may trigger a corrosion process. In fact, according to the mechanical design requirements [10], the minimum copper thickness must not be less than 50 mm. Therefore, alternative tagging solutions must be implemented. Labelling devices must be tamper indicating by a unique identity (identification) and must be able to give evidence of counterfeiting or duplication (authentication). The identification of a canister is the process of clearly distinguishing that canister from the others. The authentication, instead, is the action of proving the originality of a certain canister. The most common technologies for identification and authentication of nuclear items are described in this paper. The aim of this review is making a comparison between the different approaches, analyzing advantages and disadvantages with a view to find which techniques could be better fitting to copper canisters identification and authentication. The first part of the paper describes the most significant safeguards requirements for long term storage of nuclear spent fuel in geological repositories. Afterwards, next paragraphs give an overview of the existing technologies used for tagging of nuclear items, among which seals, Radio Frequency Identification (RFID), SERS-Active Nanoparticle Aggregates, Tungsten-based identifier, reflective laser scanning, reflective particle tag (RPT), and ultrasounds. In the end, a potential application of these technologies for copper canisters identification and authentication is discussed and results are summarized in a table. The greater part of the data collected in this paper comes from the private database of the JRC, scientific articles, nuclear authorities’ reports and companies’ websites.

## 2. Safeguards Requirements for Geological Repositories

**Figure 3:**
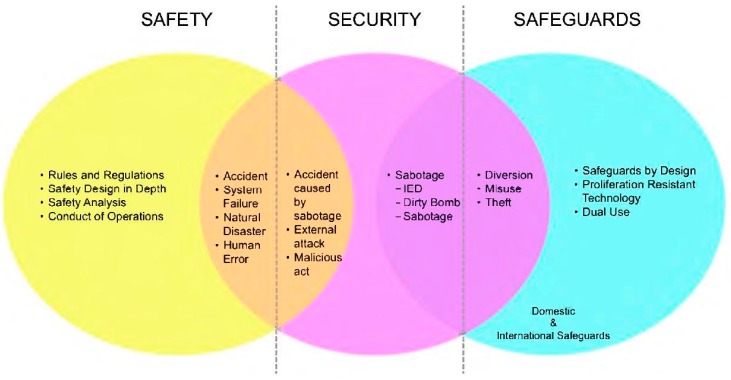
The “3S Concept” for harmonizing Safety, Security and Safeguards (IED: Improvised Explosive Device, Dual Use: technology designed or suitable for both civilian and military purposes). Source: SNL adaptation of a graphic by S. DeMuth, Los Alamos National Laboratory (LANL), ASTOR Meeting, Pori, Finland, 2013.

The IAEA and the EURATOM Safeguards approaches for encapsulation plants and geological repositories are based on integrated safeguards. The idea of integration can be resumed with the “3S concept” that shows the relationship between Safety, Security and Safeguards [11]. Figure 3 gives a graphic representation of the 3S concept. 

Repositories present several new challenges for international safeguards. Indeed, deep geological repositories will be unique among nuclear facilities. Being located entirely underground, a repository is never visible in its entirety, but can only be inspected through the use of underground mapping techniques and remote sensing methods (most commonly geophysical methods, including seismic and acoustic monitoring, ground-penetrating radar, etc.). Furthermore, unlike other nuclear facilities, the construction of additional emplacement tunnels will continue when emplacement operations will be already started. This makes inspection of a completed facility prior to operation impossible, and may add to potential difficulties with simultaneously monitoring both construction and emplacement activities, as well as with verifying design information. Another potential complicating factor is that potential changes of the design may occur when unanticipated underground conditions (e.g., faults, fractures, etc.) are encountered during construction, necessitating a change in design. Such potential for design changes during construction (and potentially during operations as well) further complicate the ability to verify design information. No other nuclear facility presents such difficulties. The principal safeguards challenges associated with both geological repositories and spent-fuel encapsulation plants include [12]:

- Verification of spent fuel at encapsulation plant prior to transport and emplacement.
- Assuring that the waste/spent fuel is emplaced as declared.
- Assuring that no waste/spent fuel is diverted before or after emplacement, including by excavating a closed and sealed repository.
- Detecting such diversion or attempts in a timely manner.
- Maintaining continuity of knowledge about waste/spent fuel destined for disposal and after disposal.
- Verifying repository design information through remote sensing methods and underground mapping, including during concurrent construction and emplacement operations.
- Applying effective containment and surveillance (C/S) measures to canisters and to the entire repository (the latter would be accomplished through remote sensing via geophysical methods, aerial and satellite imagery, etc.).

Regarding copper canisters, the IAEA and EURATOM inspectors may have the need to verify the identity of a filled spent fuel canister, before or after disposal. Therefore a system for the identification and authentication of copper canisters is required. In general, a tag consists of a pattern that can be used to identify or count specific items. In the nuclear field, tags must be well shielded against radiations and robust against duplication or counterfeiting. The main important requirements for a copper canister tagging systems are [13]:

- Large and unique tag memory.
- Security against falsification of data, errors/multiple verification.
- Environmental safety (avoid corrosion effects of e.g., copper canisters).
- Non-contact and portable/compact reader system (preferably).
- Long operation time (100 years or longer).
- Resistance to harsh environment (high radiation, temperature and humidity).
- Cost-effectiveness.
- Easy implementation and use (do not affect too much the production chain of copper canisters).
- Minimal human input (the presence of inspectors should be minimal; systems should acquire, save and transfer data automatically to inspectors’ headquarters).

Traditional tags (engraved, etched, attached, welded) are simple and practical, but may not constitute a unique identifier which could be verified by the IAEA and EURATOM. Moreover any modification to the outside of the canister may pose a risk to its long-term integrity and so must be carefully considered [14]. In fact, application of external markings may be restricted if such markings could cause corrosion or otherwise affect the integrity of the canister. Without external markings, all disposal canisters will be visually identical. Once emplaced and backfilled, canisters will be neither accessible nor visible. Even if recovered from emplacement, these canisters may be largely indistinguishable due to surface corrosion of any distinguishing external markings or other features. Furthermore a surface treatment often is susceptible to unintentional damage during normal handling, rendering them unusable.

While it is important to be able to identify individual canisters, it is also necessary to know that a canister had not been opened and any contents removed or exchanged. The copper canister external surface is not inherently tamper-indicating. The development of a suitable instrument to identify and authenticate copper canisters is still a challenge for the inspectorates. For this purpose, in the next chapters the main features of conventional tagging technologies will be briefly described, analyzing their relevance for the identification of copper canisters for long term storage of nuclear spent fuel. 

## 3. Seals

One of the oldest tagging systems for nuclear waste is the seal [15]. Seals are typically used to provide a unique identity to containers for nuclear items. The integrity of the seal can be verified over time in order to detect potential tampering attempts. The verification of a seal can be implemented with or without removing the seal from the container by checking the identity and integrity. Seals do not provide a physical protection to the sealed item but just give evidence of possible manipulations. Seals can be passive, that do not require any energy source or active, that require power to operate, like for example (Electronic Optical Sealing System (EOSS), Remotely Monitored Sealing Array (RMSA) and Active Optical Loop Seal (AOLS)).

### 3.1. Passive Sealing Systems

Passive sealing systems are very used by of the IAEA. A passive seal requires visual inspection and does not provide real-time monitoring capabilities. The most common passive seals are metal seals, adhesive seals, COBRA seals and ultrasonic seals (UOSB, USSB).

**Figure 4:**
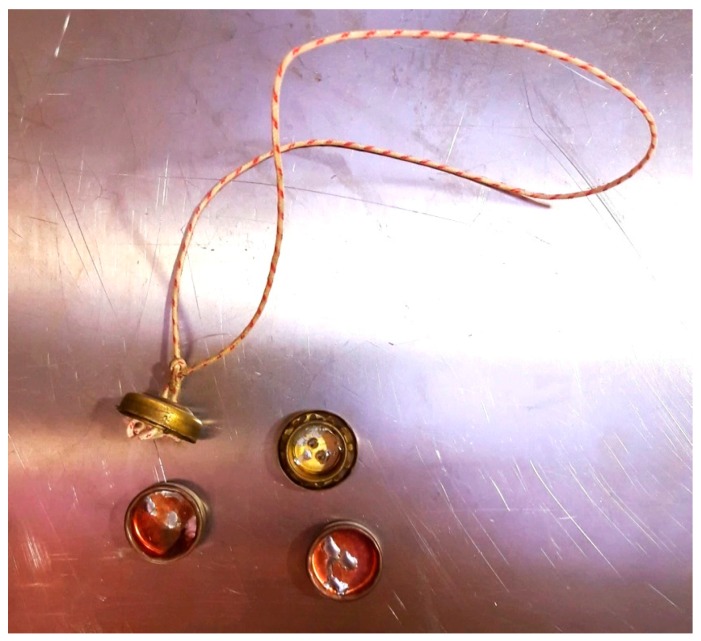
Metal cap seal.

Metal seals (CAPS) are simple and inexpensive. The seal consists of two metallic halves (caps), which can be interlocked. A piece of wire is used to attach the seal; the ends of the wire are tied inside the seal before closing it therefore the loop cannot be opened without cutting (Figure 4). Random scratches and solder smears on the inner surface of the metal cap realize a unique identity. The seal is designed in such a way that any attempt to open it would be detectable. Moreover the verification of the seal must be performed at the IAEA and EURATOM’s headquarters by comparing the images before installation and after removal. New developments aim in-situ verification by the use of the laser surface authentication method (to create a unique physical signature on the top and bottom halves of the metal seal) and the eddy current verification (to verify the wire integrity) [16]. In 2011 the IAEA began the development of a new frangible seal as a replacement for the CAPS seals. It is a two-part glass seal with random colored patterns throughout the seal body. Upon a tamper attempt, the seal body will break. The intent is that such seals can be verified in-situ for indications of tampering and for authentication. 

The adhesive seal is realized with a special material which release evidence of the detachment. Therefore this seal is intended only for temporary applications and its main advantages is the low price and the low operation, logistics and maintenance train.

The COBRA seal is composed by a fiber-optic loop that is enclosed in a seal and cut in a random way creating a unique pattern. After the seal installation, a reference image of the seal’s pattern is recorded. In such a way, during the verification of the seal, it is possible to compare the reference image with the new pattern recorded to detect potential changes [17].

**Figure 5:**
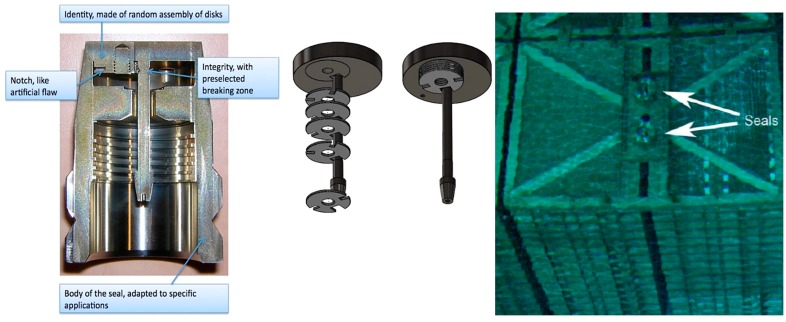
The Ultrasonic Sealing Bolt (USSB) structure and its application on underwater storage casks for nuclear items.

The Ultrasonic Optical Sealing Bolt (UOSB) and Ultrasonic Sealing System Bolt (USSB) are instead stainless steel seals that involve ultrasounds for the detection of a unique fingerprint. The USSB is used primarily for underwater applications while UOSB are applied on dry storage casks. The USSB is realized by a unique random pattern of metal discs (identity) and a frangible element (integrity) that breaks if the seal is removed from the container (Figure 5). By the ultrasonic reading of the seal, the unique pattern realized by discs and the frangible element can be detected. Patterns recorded during installation are compared with those obtained during subsequent in situ checks to verify any tampering attempts [18,19].

### 3.2. Active sealing Systems

**Figure 6:**
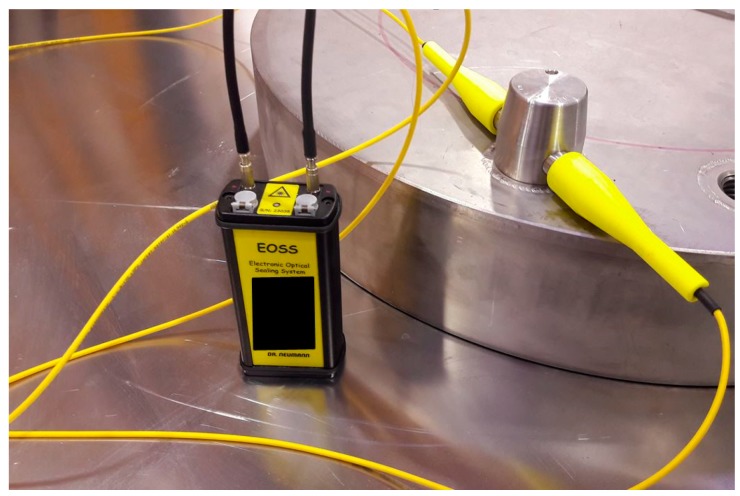
Picture of the EOSS seal connected to the UOSB seal.

The Electronic Optical Sealing System (EOSS) developed by Neumann Elecktronik GmbH is an electronic seal with a fiber-optic loop which provides evidence of opening of the seal once applied on a cask (Figure 6). The reader is realized by a laser source that verifies the integrity of the fiber-optic loop over time. The seal is reusable and high reliable thanks to the “smart” power management system. Moreover the EOSS can be remotely interrogated and provide information about status, event log, and inspection data [16,20]. 

The Remotely Monitored Sealing Array (RMSA) is developed by Mirion Technologies (Canberra) and is used for the identification of a large amount of nuclear items. The RMSA is composed by a network of electronic optical seals and a data translator to acquire data from the array. Based upon the Secure Sensor Platform (SSP) technology, the RMSA stores, forwards and communicates data by using a customized data communication protocol over a no-license, low-power radiofrequency link. The data can be stored and transferred remotely and securely (authenticated and encrypted) to the data translator that collects data from all the RMSA seals in a particular location. The seal itself is a reusable device with an expected lifespan of four to five years [21]. 

The Active Optical Loop Seal (AOLS) developed by the Joint Research Centre of the European Commission, is a new type of optical electronic seal that aims to combine the simplicity of a passive seal with the core advantages of electronic seals. It provides a continuous monitoring of the optical fiber wire and simplifies the inspector’s work. The AOLS is the first active seal with an open hardware and software architecture. This allows the inspectorates to more thoroughly assess its security and change functions to meet the wide range of containment requirements [22]. 

Concerning copper canisters identification, physical seals cannot be applied if the seal risks compromising the long-term safety performance of the canister following its emplacement in the repository. Seals could be used as a potential C/S measure for transportation of spent fuel. In fact, seals could be applied on transport casks for copper canisters assuring CoK of spent fuel during transport and shipment between the encapsulation plant and the repository site. In this case, the identification tags on the canisters could be much simpler, since the container seal ensures that the tags could not have been altered. However it depends on how CoK would be applied from the higher-level systems perspective.

## 4. Radio Frequency Identification (RFID) 

**Figure 7:**
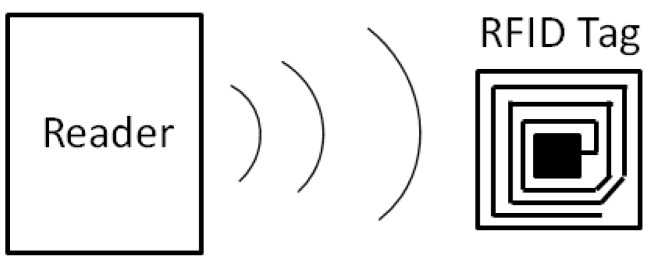
RFID tag and reader.

Comprehensive monitoring and tracking of sensitive nuclear materials can be achieved with radio frequency identification (RFID) techniques. These systems not only monitor the condition of the nuclear materials, but also track environmental and physical changes to the packages, sending real-time alerts during storage and transportation. A RFID system generally consists of tags and readers (Figure 7). The tags are attached to the objects to be identified.

RFID can be active and passive. Active tags contain a small internal power source to communicate, store and process large amounts of information in the chip. A power source is usually a lithium battery lasting less than 5 years. This makes them unsuitable for use in long-term storages. Passive tags have no battery. In order to provide power and data to the chip, they use the current in the loop antenna which is induced by the interrogating RF signal. Thus, they receive power from the reader’s antenna. The reader can be installed inside a truck trailer or storage facility and communicates with the tags through radio waves (UHF, 433.9 MHz, range of transmission up to ≈100 m). When the tag, containing several resonant circuits, enters a detection zone, the system determines the resonant frequency of each of the resonant circuits and produces a corresponding code. The system tracks the tagged objects and queries their state of health autonomously and continuously [23]. 

**Figure 8:**
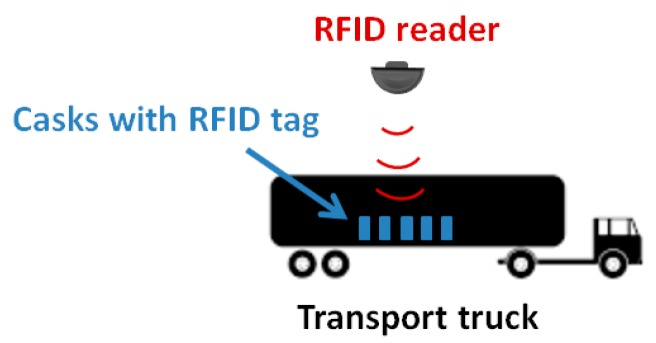
Example of an RFID tracking system.

This monitoring system (Figure 8) is then advantageous in terms of streamlined operation, improved record-keeping, enhanced safety, security and safeguards, cost savings [24]. However, many of the sensitive nuclear materials under surveillance are emitters of neutron and gamma radiation that can impede RFID working correctly. 

The implementation of RFID systems for copper canisters identification could be precluded by handling realities. In fact an exterior affixed tag may be vulnerable to damage during handling. However, a tag on the inside of a canister would not be similarly affected. As a consequence, an interior tag would not be as easily subject to intentional tampering. It would have to survive a harsh environment, certainly that from radiation, and would also need to be able to be verified from outside the canister through the thick copper wall (which is not feasible). As a result, RFID tags cannot be used due to copper shielding, but a magnetic-based technology for tagging may be possible.

## 5. SERS-Active Nanoparticle Aggregates 

**Figure 9:**
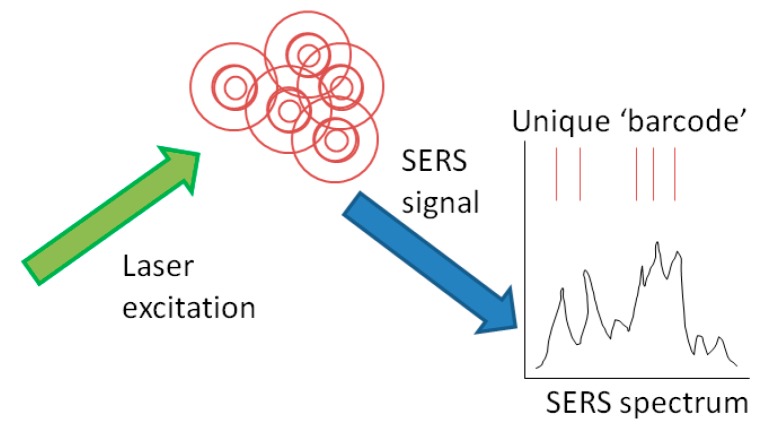
Laser excitation of SANAs to provide a spectroscopic barcode.

The Surface Enhanced Raman Scattering (SERS) response from SANAs (SERS-Active Nanoparticle Aggregates) provides a unique identifier or signature for tagging applications. SANAs are formed from gold or silver nanoparticles in the 40–80 nm size range. A chemical ‘dye’ is attached to the nanoparticle surface and the nanoparticles are aggregated into ensembles. The dye provides individual identifying information to the aggregate, which is finally coated in a glass layer to provide environmental stability. The completed SANA can be physically embedded in or chemically surface-mounted to tag or seal materials. Upon excitation by a laser (Figure 9), a SANA returns a spectroscopic signal unique to the dye, or combination of dyes, absorbed by the SANA metal surface. Depending on the application, the signal can be reduced to a simple barcode, or examined in complex detail to provide a high security signature that is cost prohibitive to replicate. 

SANA-based tags incorporate the fundamental advantages of most passive tagging and sealing technologies. The system will be inexpensive due to low material costs for the tags, and shared verification hardware. No on-site power sources or infrastructure will be required. No RF, Wi-Fi, or other networking technologies will be required. The SANAs signature can be extensively varied to provide a library of unique tag systems. However the apparent weakness in this approach is the possibility that the identity of the chemical dye could be determined, leading to the direct preparation of a counterfeit batch of SANAs. Moreover silver and gold nanoparticles are known to suffer structural degradation over long periods, or during adverse environmental exposure, then a silica (or glass) coating should be applied to improve the stability of the fingerprint. In order to increase the tag security, SANAs could be integrated with Reflective Particle Tags (RPT) realizing a highly secure, tamper evident and traceable passive tagging system [25]. 

The use of SERS-Active Nanoparticle Aggregates for copper canisters identification requires the application of a tag or a seal on the surface of the container. As already discussed for the RFID technology, the presence of an exterior tag could be a problem during handling. However if the tag only needs to last until final emplacement, then an affixed tag could be used, and removed if necessary at emplacement. Moreover, SERS-Active Nanoparticle Aggregates tags cannot be placed inside canisters because the laser used for the reading of the fingerprint cannot penetrate through the 50 mm of copper thickness due to the high attenuation. 

## 6. Reflective Particle Tags (RPT)

The Reflective Particle Tag (RPT) is a next generation of tag technology developed with the goal of providing both a unique identifier and visual evidence of tampering. They are resin tags infused with reflective crystals that are placed over sensitive seals. The goal is developing a verification routine based on image processing that verifies the original set of images of an RPT, against a set taken at a later time. Each set contains images taken by illuminating the RPT from a number of orientations, creating different views due to crystal reflections. 

**Figure 10:**
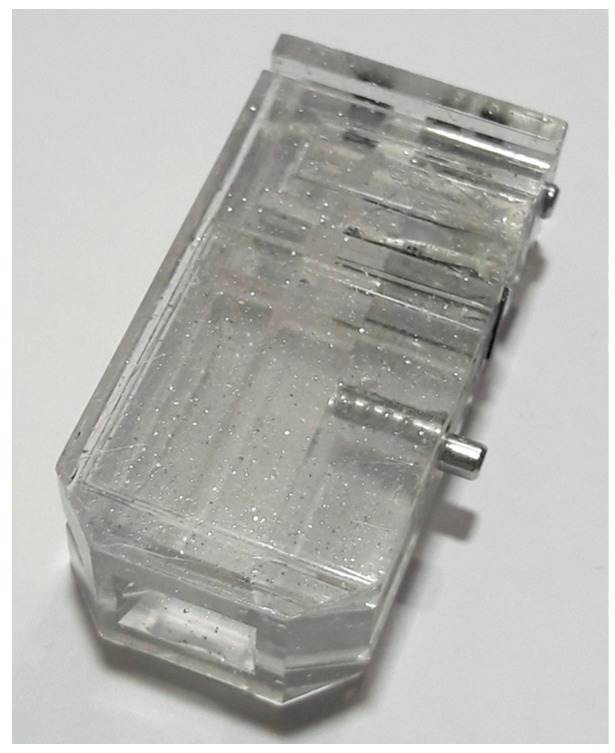
Reflective particles tag on a COBRA seal.

The tag (Figure 10) consists of reflective particles mixed in a transparent adhesive matrix, which is applied to the surface of the item to be identified and then cured. A reader consisting of a number of lights and some means of recording an image is used to read the patterns formed by the reflectors in the tag. Comparing images of the tag to images taken when the tag was applied verifies the identity of the tag and therefore the item [26]. 

The RPT architecture has proven resistant to counterfeiting and removal without detection. Furthermore, the tag requires no power, and is stable through temperature extremes, rough handling, and years of service. Concerning copper canisters identification, if affixed tags could be used, the reflective particle tag (RPT) may be a possible option. However locating RPT and SANAs tags inside the canister is not very easy. Furthermore, the reader system suffers from image degeneration and irregular calibrations. Moreover if the RPT tag is installed on a seal placed on the surface of a canister, it could trigger a corrosion process, undercutting the long term stability of the canister structure [27].

## 7. Reflective Laser Scanning

**Figure 11:**
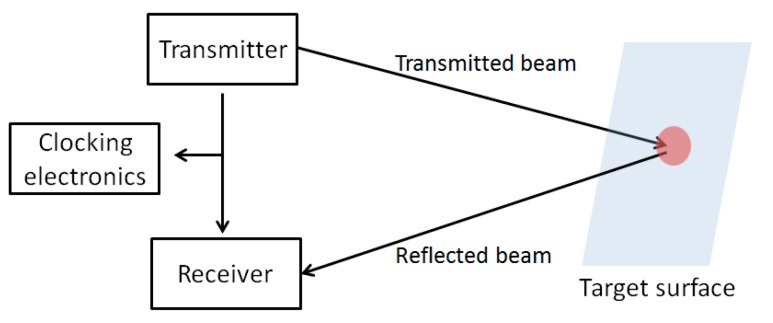
Reflective laser scanning system.

During transportation and storage of nuclear items, their location and security can be verified by a low power laser scanning system and retro-reflective material used as tags. An identification tag comprises a retroreflective substrate for reflecting incident light beams, and patterned indicia for selectively reducing retro reflectivity of the retroreflective substrate. The unique identity is due to the presence of a non-reflective material on the retroreflective surface of the tag. Tags affixed to the items can be monitored from a fixed observation point by a laser scanning system having a rotating mirror with ultra-precise angular resolution (Figure 11). If a tag is missing or it has moved, the system detects this change. 

Considering a potential application of this system for copper canisters identification, the same set of problems observed for RFID systems can be found. Unlike RFID, this method of information transfer is virtually impossible to intercept or falsify [28]. However tags could be removed and reattached or even duplicated, without alerting the system. In order to avoid any risks of failure, having redundancy could be effective but also expensive in terms of costs and resources. Moreover reflective laser scanning is most useful for long term static storage and not for monitoring during transport of canisters. Indeed the application of tags on the external surface could be a problem during handling of canisters.

## 8. Tungsten-Based Identifier 

**Figure 12:**
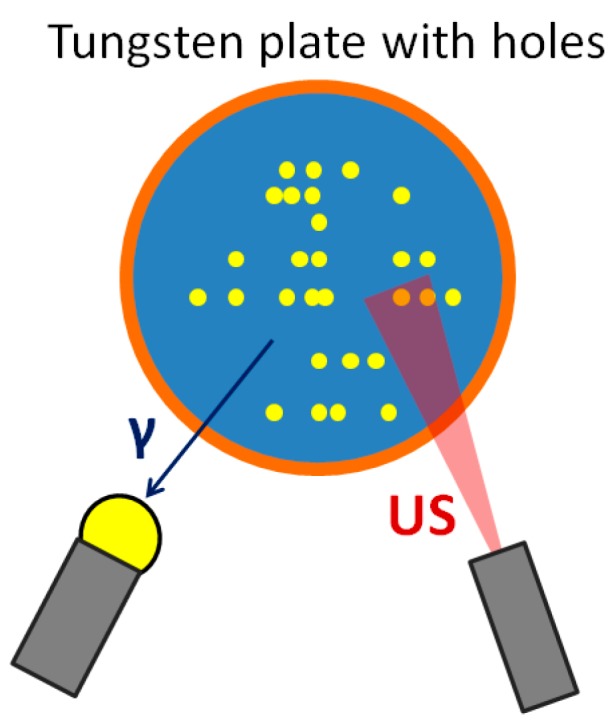
Set-up of the Tungsten-based identifier.

The Tungsten-based identifier has been specifically developed for the identification of copper canisters for spent nuclear fuel. This method envisages the realization of a series of holes on a tungsten plate, reproducing a unique binary code. The tungsten insert is located inside copper canisters, between the top iron lid and the copper lid. The configuration of holes is machined in correspondence of the spent fuel assemblies in order to detect the gamma rays. Because of the strong collimation of gamma rays due to tungsten attenuation features, the unique code realized on the tungsten plate can be acquired by a gamma rays detector placed above the copper lid. In particular, the change of the gamma-counting rate in the reader gives evidence of the holes’ disposition and then the unique fingerprint. More than a binary code can be realized on the plate to guarantee redundancy and an ultrasonic transducer can be used to confirm the location of holes on the plate (Figure 12). Depending on the source of gamma rays, this approach can involve passive tags (intrinsic source) or active tags (artificial source). 

The active tag is realized by α-emitting isotopes with a high energy that create a unique pattern, avoiding non-uniformities of the radiation background. Nevertheless, this type of tags is difficult to implement in the real case. In fact, the presence of radioisotopes implies the installation of a devoted laboratory at the encapsulation plant and increase the radioactivity level of copper canisters. On the contrary, the practical implementation of passive tags is more realistic because they need only the radiation of the spent fuel. 

This way of identification of the copper cask does not impair the integrity of the cask and it offers a way for the information about the spent nuclear fuel to be read up to a few thousand years. Moreover, differently from the other technologies that involve tags attached on the surface, this method avoids any problems related to canisters handling. Nevertheless, the tag is artificially prepared and then it could be reproduced. The only aspect that can contribute to define this solution secure is the high cost of replication. [29,30]. In addition, the use of ultrasounds to reveal discontinuities in the tungsten layer is critical due to the coupling between the copper lid and the tungsten plate.

## 9. Ultrasonic Systems

**Figure 13:**
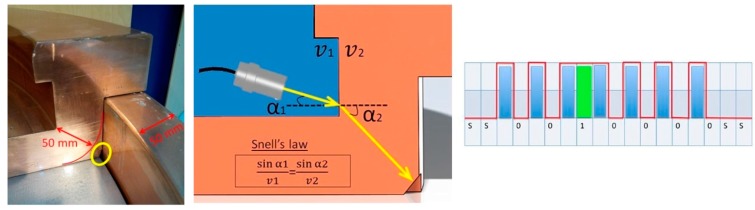
The ultrasonic investigation of chamfers, artificially machined on canisters’ lid to create a unique identification fingerprint.

The identification of copper canisters could be realized by the ultrasonic acquisition of a double fingerprint: artificial and natural [31]. The first signature is created by milling a series of chamfers on the inner surface of the copper lid before closure onto the canister by Friction Stir Welding (FSW). Chamfers are arranged around the lid circumference, in an area (yellow circle in Figure 13) where the copper thickness is higher than 50 mm, which is the minimum thickness to fulfill mechanical design requirements. The investigation of chamfers is accomplished by an ultrasonic transducer that rotates 360° around the circumference with an inclination according to the Snell’s law. The ultrasonic amplitude response acquired by the probe, represents a unique binary code, strictly related to chamfers configuration [32]. The machining of chamfers on the lid guarantees a long term fingerprint for copper canisters and can be easily included within the machining process of the lid during the production of canisters. 

**Figure 14:**
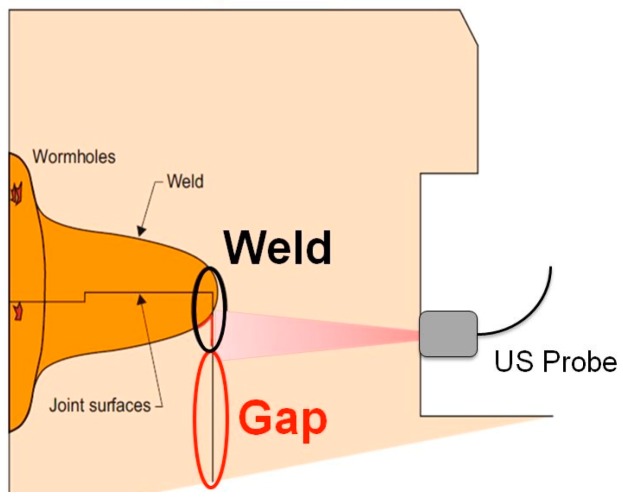
Ultrasonic investigation of the internal gap between lid and canister after friction stir welding.

In case of counterfeit of canisters, their disposition can be easily duplicated and ultrasonic inspections cannot reveal any change. Therefore the investigation of the welding area between lid and canister could be useful to assess the authenticity of the container. In particular, ultrasounds can be used to acquire the fingerprint related to the variation of the internal gap between lid and tube after FSW (Figure 14). The ultrasonic amplitude response acquired with an ultrasonic probe at 10 MHz of frequency could be used as a unique signature for each canister [33]. First experimental tests carried out on copper lid already welded onto canisters showed the presence of several points of interest in the fingerprint that could be used for the authentication of each canister. Therefore the identification of copper canisters could be ensured by an artificial fingerprint created by chamfers and the authentication by a natural fingerprint due the welding area. The angular matching of these two signatures, realize a third fingerprint, more robust and reliable. 

Differently to all the other tagging methods, the ultrasonic approach is the only one which cannot be reproduced because is based on a natural fingerprint. In fact, the structure of the material during the welding process could be altered in a random way and a high frequency inspection by ultrasound can reveal it. Moreover the ultrasonic amplitude response is strictly related to the attenuation properties of copper whose grain structure could vary a lot creating different patterns for each canister. 

## 10. Comparison between Tagging Technologies 

Following the description of the most common tagging technologies for nuclear items, their potential implementation for copper canisters identification and authentication is discussed in this chapter. Table 1 shows if each technology meets or not all the requirements illustrated in Chapter 2. Based on the list of requirements and the results obtained, a comparison between tagging systems can be done:

- *Large and unique tag memory*: all the tags respect the requirement. This specification is at the base of the definition of a tagging system and then it is necessary but not sufficient. The labelling system should enable fully unique identification of the canister content in a manner consistent with permanent records of the storage or repository.
- *Security against falsification of data*: this requirement, in addition with the previous, is of upmost importance for copper canisters. It implies a strong reliability of the tagging system, as well as a strong immunity to falsification or error. No technology satisfies this specification, except ultrasonic reading of welding/grain “anomalies”. In fact, the fingerprint of the welding area is strictly related to material/welding properties, typical of each container and then not reproducible.
- *Environmental safety*: only the Tungsten-based identifier and ultrasounds could avoid problems with copper corrosion. In fact the labelling system should avoid corrosion effects of canisters which can be induced in the long-term run, thus for instance avoiding leakage of spent fuel waste components later on. For these reasons, the application of all the technologies which imply the use of seals on the canister cannot be considered.
- *Non-contact reader system*: each tag could implement a non-contact reader. This minor requirement aims to preserve the copper canister external surface and above all to protect the reader from the high radiation level of spent fuel.
- *Long operation time*: the operating time should be at least to the end of the deposition of the last canister which is estimated to last 30 years. As a consequence, Radio Frequency Identification (RFID), SERS-Active Nanoparticle Aggregates and Reflective Particle Tags (RPT) do not satisfy this requirement. RFID systems have an operation time less than 7 years related to the interference of the metallization layer with RF signal. The other two tags, instead, suffer from image degradation over time.
- *Resistance to harsh environment*: the presence of a high radiation level could compromise the mode of operation of the tagging system. Moreover the presence of high temperatures could affect the electronics of reader devices or reduce the accuracy of measures. There are no experimental results available on the impact of a harsh environment on the effectiveness and longevity of tagging systems. Therefore from this review the authors envisaged that future studies must be carried out to verify the resistance of each technology.
- *Cost-effectiveness*: the cost is not a relevant parameter for the Safeguards of spent fuel. However certain technologies, such as SERS-Active Nanoparticle Aggregates, Reflective Particle Tags (RPT), Reflective laser scanning and Tungsten-based identifier have a high cost for duplication which is deterrent for duplication.
- *Easy implementation and use*: the implementation of all the tagging systems is not very easy because most of them require the presence of a seal or an adhesive tag at least, which makes difficult the handling of canisters.
- *Minimal human input*: all the reading of tags could be potentially automated and then would not require any human input once applied on canisters. Data could be acquired by the system, saved and transferred remotely to the inspectorates’ headquarters.

## 11. Conclusions

In order to keep the Continuity of Knowledge of spent nuclear fuel and guarantee a sufficient level of security against misuse or counterfeiting of copper canisters, a robust identification and authentication technology must be implemented. Following the analysis of Safeguards requirements for geological repositories and copper canisters in detail, an overview of existing tagging technologies is reported in the previous chapters. In order to better identify which technique could be the most suitable for copper canisters identification and authentication, a comparison between technologies is accomplished. A table is fulfilled to better visualize if requirements were satisfied or not by each tagging system. In conclusion, the introduction of copper canisters for long term geological repositories has brought the necessity to develop new tagging methods based on modern technologies in the field of electronics, optics or computer science. As a result of this study, only the Tungsten-based method and ultrasounds could satisfy the desire to avoid any introduction of additional or unforeseen corrosion effects on the copper canisters. However, the ultrasonic system better meets all the requirements, especially regarding the ability to authenticate containers. In fact, all the tagging systems analyzed are based on a tag artificially created, while the fingerprint due to the welding area is a natural fingerprint, related to the material structure of each copper canister, which can’t be reproduced. 

## 12. Patents

F. Littmann, “Ultrasonic identification and authentication of containers for hazardous materials”, European Patent 16,166,465, 21 April 2016.

## Figures and Tables

**Table 1:** List of existing tagging technologies that fulfill or not the requirements for copper canisters identification.

Requirements	Existing Tagging Technologies						
	Seals	Radio Frequency Identification (RFID)	SERS-Active Nanoparticle Aggregates	Reflective Particle Tags (RPT)	Reflective Laser Scanning	Tungsten-Based Identifier	Ultrasonic Systems
Large and unique tag memory	Yes	Yes	Yes	Yes	Yes	Yes	Yes
Security against falsification of data	Yes	No	No	No	No	No	Yes
Environmental safety	No	No	No	No	No	Yes	Yes
Non-contact reader system	Yes	Yes	Yes	Yes	Yes	Yes	Yes
Long operation time	Yes	No	No	No	Yes	Yes	Yes
Resistance to harsh environment	Unknown	Unknown	Unknown	Unknown	Unknown	Unknown	Unknown
Cost-effectiveness	Yes	Yes	No	No	No	No	Yes
Easy implementation and use	No	No	No	No	No	Yes	Yes
Minimal human input	Yes	Yes	Yes	Yes	Yes	Yes	Yes
